# Identification of clinical factors associated with severe dengue among Thai adults: a prospective study

**DOI:** 10.1186/s12879-015-1150-2

**Published:** 2015-10-14

**Authors:** Vipa Thanachartwet, Nittha Oer-areemitr, Supat Chamnanchanunt, Duangjai Sahassananda, Akanitt Jittmittraphap, Plengsakoon Suwannakudt, Varunee Desakorn, Anan Wattanathum

**Affiliations:** Department of Clinical Tropical Medicine, Faculty of Tropical Medicine, Mahidol University, 420/6 Rajvithi Road, Bangkok, 10400 Thailand; Pulmonary and Critical Care Division, Department of Medicine, Phramongkutklao Hospital, 315 Rajvithi Road, Bangkok, 10400 Thailand; Information Technology Unit, Faculty of Tropical Medicine, Mahidol University, 420/6 Rajvithi Road, Bangkok, 10400 Thailand; Department of Microbiology and Immunology, Faculty of Tropical Medicine, Mahidol University, 420/6 Rajvithi Road, Bangkok, 10400 Thailand; Hospital for Tropical Diseases, Faculty of Tropical Medicine, Mahidol University, 420/6 Rajvithi Road, Bangkok, 10400 Thailand

**Keywords:** Dengue fever, Lactate, Thailand, Predictive factors

## Abstract

**Background:**

Dengue is the most common mosquito-borne viral disease in humans. Recently, there has been an epidemic shift of dengue from mainly affecting children to affecting more adults with increased severity. However, clinical factors associated with severe dengue in adults have varied widely between studies. We aimed to identify the clinical factors associated with the development of severe dengue according to the World Health Organization (WHO)’s 2009 definition.

**Methods:**

We conducted a prospective study of adults with dengue admitted to the Hospital for Tropical Diseases in Bangkok, Thailand, from October 2012 to December 2014. Univariate and stepwise multivariate logistic regression analyses were performed.

**Results:**

Of the 153 hospitalized patients with confirmed dengue viral infections, 132 (86.3 %) patients had non-severe dengue including dengue without warning signs (7 patients, 5.3 %) and dengue with warning signs (125, 94.7 %). The rest (21, 13.7 %) had severe dengue including severe plasma leakage (16, 76.2 %), severe organ involvement (16, 76.2 %), and severe clinical bleeding (8, 38.1 %). Using stepwise multivariate logistic regression, clinical factors identified as independently associated with the development of severe dengue were: (1) being >40 years old (odds ratio [OR]: 5.215, 95 % confidence interval [CI]: 1.538–17.689), (2) having persistent vomiting (OR: 4.817, CI: 1.375–16.873), (3) having >300 cells per μL of absolute atypical lymphocytes (OR: 3.163, CI: 1.017–9.834), and (4) having lactate levels ≥2.0 mmol/L (OR: 7.340, CI: 2.334–23.087). In addition, increases in lactate and absolute atypical lymphocyte levels corresponded with severe dengue (*p* < 0.05).

**Conclusions:**

Our study identified several clinical factors independently associated with the development of severe dengue among hospitalized adults with dengue. This can aid in the early recognition and prompt management of at-risk patients to reduce morbidity and mortality.

## Background

Dengue is caused by four dengue virus serotypes (DENV 1–4) and it is the most rapidly spreading mosquito-borne viral disease in humans [1]. Annually, approximately 50–100,000 000 people contract dengue viral infections worldwide and an estimated 500,000 people with dengue hemorrhagic fever (DHF) require hospitalization [2]. The mortality rate of dengue is approximately 20,000 deaths per year with a morbidity of 264 disability-adjusted life years per million people per year [1]. During the past decades, the emergence of dengue has been reported throughout the world with a 30-fold increase in global incidence and severity. Approximately 75 % of the global populations exposed to dengue reside in the Asia-Pacific region [1].

Previous reports have shown an epidemic shift of dengue from mainly affecting children to now affecting more adults [2–4]. Children and adults with dengue differ in both clinical manifestations and laboratory findings [5, 6], and the incidence of “severe dengue” according to the WHO's 2009 definition has increased among adults [2, 7]. In 2009, the WHO introduced a new dengue case definition to emphasize clinical management and to increase the sensitivity and specificity of severe-dengue diagnosis [8]. A recent systemic review of the application of the WHO’s 2009 definition showed a sensitivity of 59–98 % and a specificity of 41–99 % for identifying severe dengue [9].

In Thailand, the number of adults with dengue has increased dramatically in recent decades [1, 4]. According to the WHO’s 2009 definition, approximately 27.9 % of hospitalized Thai adults with dengue had severe dengue from 2006 to 2010 [10]. Although several studies have been conducted, the clinical factors associated with severe dengue in adults have varied widely due to (1) the differences in applying the definitions of severe dengue, by using either the WHO’s 1997 definition or the WHO’s 2009 definition, (2) differences in the study designs, and (3) differences in the data analyses [10–14]. Thus, these studies have reported widely varying results of the clinical factors associated with severe dengue, including having secondary dengue infections, age >37 years, male gender, presence of intense asthenia, presence of abdominal pain, presence of cough, presence of bleeding, a mean arterial pressure (MAP) less than 80 mmHg, an increase in hematocrit levels to more than 2 % over the reference range (adjusted for gender), low lymphocyte level, higher aspartate aminotransferase (AST) or alanine aminotransferase (ALT) levels by more than 3 times, and low total protein level [10–14]. Currently, the WHO’s 2009 definition has been widely used for the diagnosis of severe dengue [8, 9]. Therefore, we conducted a prospective study among adults admitted to the hospital with dengue in order to determine the clinical factors associated with the development of severe dengue using the WHO’s 2009 definition.

## Methods

### Study design and population

This prospective study was conducted at the Hospital for Tropical Diseases, Faculty of Tropical Medicine, Mahidol University in Bangkok, Thailand. Patients admitted to the hospital between October 2012 and December 2014 who met the study criteria were approached for participation. The study’s inclusion criteria were: (1) adults at least 15 years old; (2) presenting with clinical criteria for dengue defined as acute fever with at least two of the following symptoms: headache, retro-orbital or ocular pain, myalgia, arthralgia, rash, a positive tourniquet test (defined as the presence of ≥20 petechiae per 1 square inch), or leukopenia (defined as a white blood cell count [WBC] <5.0 × 10^3^ cells per μL); and (3) having a confirmed dengue viral infection, defined as positive tests of either (a) viral nucleic acid using reverse-transcriptase polymerase chain reaction (RT-PCR) from serum samples on admission or (b) specific dengue IgM and IgG antibodies using enzyme-linked immunosorbent assays (ELISA) from serum samples on admission and at least two weeks later. Patients with a history of underlying medical illness or mixed infection, or who were pregnant, were excluded from this study.

The laboratory investigations including complete blood count, blood chemistries, and peripheral venous lactate were performed at patient admission. All dengue patients in this study received standard care according to WHO guidelines [8]. Patient data including baseline characteristics, clinical parameters, and laboratory findings were recorded in a pre-defined case-report form. Severity of dengue was summarized on the date of discharge according to the WHO’s 2009 definition [8].

### Case definition of dengue

According to the WHO’s 2009 dengue case definition [8], dengue patients were classified into *non-severe dengue* and *severe dengue* based on clinical and laboratory criteria. Patients with non-severe dengue were sub-categorized into two groups depending on the presence or absence of warning signs. Non-severe dengue without warning signs was defined as having acute fever with at least two of the following criteria: nausea, vomiting, rash, myalgia, arthralgia, a positive tourniquet test, or leukopenia. Warning signs included: (1) abdominal pain, (2) persistent vomiting (vomiting with signs of dehydration), (3) clinical fluid accumulation, (4) lethargy, (5) liver span >15 cm, (6) bleeding from mucosal areas including nose, gums, gastrointestinal tract or vagina, or (7) an elevated hematocrit, >2 % above the reference range for a healthy Thai adult adjusted for gender with platelet counts ≤100 × 10^3^ per μL. Severe dengue was classified as having: (1) severe plasma leakage, defined as plasma leakage with shock or respiratory distress (respiratory rate ≥24 breaths/min with oxygen saturation <95 % in room air and/or requiring oxygen therapy), (2) severe clinical bleeding, defined as spontaneous bleeding from mucosal areas that necessitates a blood transfusion or bleeding in vital organs, (3) severe organ involvement, defined as AST >1000 IU/L and/or ALT >1000 IU/L, serum creatinine ≥3 times above baseline, myocarditis, and/or encephalitis.

### Reverse-transcriptase Polymerase Chain Reaction (RT-PCR)

Dengue viral RNA in patient sera was extracted using two rounds of PCR as described by Lanciotti et al. [15] with modifications according to Reynes et al. [16]. Samples were extracted using a PureLink® Viral RNA/DNA Mini Kit (Invitrogen™, USA) according to the manufacturer’s instructions.

### Serology for dengue viral infection

All sera were tested with four separate assays of IgM and IgG antibodies for dengue viruses and Japanese encephalitis virus (JEV) using capture ELISA as described by Innis et al. [17]. In order to differentiate dengue viral infections from other flavivirus infections, the ratio of dengue IgM to JEV IgM was calculated. A ratio ≥1.0 was defined as dengue virus infection whereas a ratio <1.0 was defined as other flavivirus infection. To assess primary and secondary dengue infections, the ratio of dengue IgM to dengue IgG was calculated. A ratio ≥1.8 was considered a primary dengue infection whereas a ratio <1.8 was considered to indicate a secondary dengue infection.

### Measurement of peripheral venous lactate

Blood samples for peripheral venous lactate were collected from veins of upper extremities without the use of a tourniquet. We placed 2 ml of blood in a vacutainer containing sodium fluoride and immediately placed the sample on ice. Samples were sent to the laboratory and lactate levels were tested within 10 min of being drawn using a colorimetric assay (Roche/Hitashi cobas c systems, USA) according to the manufacturer’s instructions. The coefficient of variation in lactate assay levels in the central laboratory of the Hospital for Tropical Diseases is estimated to be 1.1 %.

### Sample-size calculation

We estimated the required sample size for each potentially associated factor and used the highest estimated number. The required sample size for this study was estimated using the Power and Sample Size Program, version 3.0, 2009 [18]. Based on our previous study, the rate of developing severe dengue among hospitalized adults with dengue who had MAP ≥80 mmHg was 0.25 [10]. If the true relative risk of developing severe dengue among patients with MAP <80 mmHg was double that of patients with MAP ≥80 mmHg then the study required a 1:2 ratio of patients with MAP <80 mmHg to patients MAP ≥80 mmHg. We needed to study 48 patients with MAP <80 mmHg and 96 patients with MAP ≥80 mmHg to be able to reject the null hypothesis that the relative risk between these groups was equal to 1 with probability (power) of 0.85. The type I error probability associated with this test was 0.05. Thus, we needed to study a minimum of 144 patients with dengue.

### Statistical analyses

Data were analyzed using SPSS for Windows 18.0 (IBM Corp., Chicago, IL). Numerical variables were tested for normality using Kolmogorov-Smirnov tests. Variables with non-normal distributions were summarized with medians and inter-quartile ranges (IQR) and compared using Mann–Whitney *U* tests. Categorical variables were expressed as frequencies and percentages and then analyzed with chi-square tests or Fisher’s exact tests, as appropriate. A univariate logistic regression was performed with each potential factor included as an independent variable and the presence or absence of severe dengue infection as the dependent variable. Any variable with a *p*-value less than 0.2 was considered potentially significant and then further analyzed in a stepwise multivariate logistic regression using the backward selection method for determining significant independent factors. All tests of significance were two-sided with *p* < 0.05 indicating statistical significance.

### Ethics statement

This study was approved by the Ethics Committee of the Faculty of Tropical Medicine, Mahidol University, Bangkok, Thailand. The procedure indicated by the Standards for the Reporting of Observation Studies in Epidemiology (STROBE) was followed [19]. Written informed consent was obtained from all the patients or the patients’ guardians in cases where the patients were less than 18 years of age. Data were made anonymous before analyses.

## Results

**Fig. 1:**
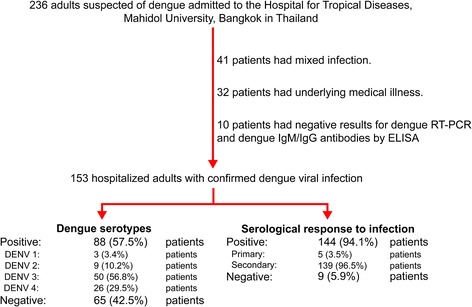
Flow diagram for recruitment and assessment of study patients

From October 2012 to December 2014, there were 236 patients admitted to the Hospital for Tropical Diseases in Bangkok, Thailand, with suspected dengue infections. Of these, 83 patients were excluded from our study because they had a mixed infection (41 patients), a history of underlying medical illness (32 patients), or negative results for dengue in their RT-PCR and IgM/IgG antibody tests (10 patients). Thus, a total of 153 hospitalized patients with confirmed dengue viral infections were recruited to the study. Among these participants, dengue serotypes were identified using RT-PCR in 88 (57.5 %) patients, indicating DENV 1 (3 patients, 3.4 %), DENV 2 (9, 10.2 %), DENV 3 (50, 56.8 %), and DENV 4 (26, 29.5 %). Dengue IgM/IgG antibodies were detected by ELISA in 144 patients (94.1 %) indicating a primary dengue infection in 5 patients (3.5 %) and secondary dengue infection in 139 patients (96.5 %; see Fig. 1).

Of the 153 patients with a confirmed dengue infection, 132 (86.3 %) had non-severe dengue including dengue without warning signs (7 patients, 5.3 %) and dengue with warning signs (125 patients, 94.7 %). The remaining 21 patients (13.7 %) had severe dengue. Of the 21 patients with severe dengue, 16 (76.2 %) had severe plasma leakage, 16 (76.2 %) had severe organ involvement, and 8 (38.1 %) had severe clinical bleeding. Of the 16 patients with severe plasma leakage, 12 (75.0 %) had plasma leakage with shock and 8 (50.0 %) had plasma leakage with respiratory distress. Of the 16 patients with severe organ involvement, 9 (56.2 %) had AST >1000 IU/L and/or ALT >1000 IU/L, 9 (56.2 %) had serum creatinine ≥3 times above baseline, 1 (6.2 %) had myocarditis, and 1 (6.2 %) had encephalitis.

### Comparison between patients with severe and non-severe dengue

Regarding the baseline characteristics of study patients, the development of severe dengue was significantly associated with (1) patient age being >40 years (9 [42.9 %] patients with severe dengue vs. 19 [14.4 %] patients with non-severe dengue; *p* = 0.004) and (2) patients receiving nonsteroidal anti-inflammatory drugs (NSAIDs) or cyclooxygenase II (COX II) inhibitors before admission (6 [28.6 %] patients with severe dengue vs. 13 [9.8 %] patients with non-severe dengue; *p* = 0.027). Other patient characteristics such as gender, residential area, and receiving acetaminophen before admission were similar between patients with severe and non-severe dengue (see Table 1).

**Table 1:** Patient characteristics. Baseline characteristics and clinical parameters at admission of 153 hospitalized adults with either severe or non-severe dengue according to the WHO’s 2009 definition

Characteristics	Severe dengue (*n* = 21)	Non-severe dengue (*n* = 132)	*p*-value
	# (%)	# (%)	
*Baseline characteristics*			
Age			0.004
≤ 40 years	12 (57.1)	113 (85.6)	
> 40 years	9 (42.9)	19 (14.4)	
Gender			0.771
Male	10 (47.6)	71 (53.8)	
Female	11 (52.4)	61 (46.2)	
Residential area			1.000
Bangkok	18 (85.7)	109 (82.6)	
Outside Bangkok	3 (14.3)	23 (17.4)	
Medication before admission			
Acetaminophen	20 (95.2)	123 (93.2)	1.000
NSAIDs or COX II inhibitors	6 (28.6)	13 (9.8)	0.027
*Clinical parameters*			
Vital signs			
Temperature, median (IQR), °C	37.8 (37.0–39.0)	38.4 (37.8–39.2)	0.162
HR, median (IQR), beats/min	82 (68–90)	79 (66–88)	0.176
MAP, median (IQR), mmHg	83 (70–92)	86 (78–92)	0.561
PP, median (IQR), mmHg	32 (26–45)	38 (33–45)	0.058
History and physical examinations			
Myalgia	20 (95.2)	120 (90.9)	1.000
Lethargy	19 (90.5)	101 (76.5)	0.251
Headache	18 (87.5)	113 (85.6)	1.000
Fever ≥4 days	17 (81.0)	84 (63.6)	0.191
Tourniquet test positive	16 (76.2)	111 (84.1)	0.359
RR ≥24 breaths/min	15 (71.4)	112 (84.8)	0.206
Mucosal bleeding	14 (66.7)	48 (36.4)	0.017
Skin bleeding	13 (61.9)	28 (21.2)	<0.001
Liver span > 15 cm	12 (57.1)	19 (14.4)	<0.001
Retro-orbital pain	11 (52.4)	90 (68.2)	0.241
Decrease breath sound	11 (52.4)	6 (4.5)	<0.001
Arthralgia	10 (47.6)	37 (28.0)	0.120
Rash	10 (47.6)	61 (46.2)	1.000
Abdominal pain	9 (42.9)	53 (40.2)	1.000
Persistent vomiting	9 (42.9)	15 (11.4)	0.001
Diarrhea	5 (23.8)	44 (33.3)	0.537

Abbreviations: *NSAIDs* nonsteroidal anti-inflammatory drugs, *COX II* cyclooxygenase II, *IQR* interquartile range, *HR* heart rate, *MAP* mean arterial pressure, *PP* pulse pressure, *RR* respiratory rate

The majority of symptoms at admission were similar between patients with severe and non-severe dengue with the following exceptions, which were more frequent among patients with severe dengue than those with non-severe dengue (see Table 1): (1) mucosal bleeding (14 [66.7 %] patients with severe dengue vs. 48 [36.4 %] patients with non-severe dengue; *p* = 0.017), (2) skin bleeding (13 [61.9 %] patients with severe dengue vs. 28 [21.2 %] patients with non-severe dengue; *p* < 0.001), (3) liver span >15 cm (12 [57.1 %] patients with severe dengue vs. 19 [14.4 %] patients with non-severe dengue; *p* < 0.001), (4) decreased sound breathing (11 [52.4 %] patients with severe dengue vs. 6 [4.5 %] patients with non-severe dengue, *p* < 0.001), and (5) persistent vomiting (9 [42.9 %] patients with severe dengue vs. 15 [11.4 %] patients with non-severe dengue; *p* = 0.001).

Laboratory findings of patients at admission are shown in Table 2. Most laboratory results among patients with severe and non-severe dengue were similar with the exceptions of platelet counts and albumin, which were both significantly lower among patients with severe dengue compared to those with non-severe dengue (*p* < 0.001). However, patients with severe dengue had significantly greater: WBC (*p* = 0.034), absolute atypical lymphocyte count (*p* = 0.011), lactate level (*p* < 0.001), AST level (*p* < 0.001), and ALT level (*p* = 0.003). When patients were categorized based on the upper or lower limits of the reference ranges, patients with severe dengue were significantly more likely to fall into the following categories: absolute lymphocyte count >2000 cells per μL (*p* = 0.036), absolute atypical lymphocyte count >300 cells per μL (*p* = 0.002), platelet count ≤100 × 10^3^ per μL (*p* = 0.031), lactate level ≥2.0 mmol/L (*p* < 0.001), albumin level <3.5 g/dL (*p* < 0.001), AST level >120 IU/L (*p* = 0.017), and ALT level >120 IU/L (*p* = 0.032). The median (IQR) duration of hospitalization among patients with severe dengue were significantly longer than those with non-severe dengue (4.8 [2.9–9.8] vs. 3.7 [2.7–4.8] days; *p* = 0.047).

**Table 2:** Laboratory test results. Laboratory parameters on admission of 153 hospitalized adults with dengue by dengue severity according to the WHO’s 2009 definition

Characteristics	Severe dengue (*n* = 21)	Non-severe dengue (*n* = 132)	*p*-value
	# (%)	# (%)	
*Confirmation tests for dengue*			
Known dengue RT-PCR (*n* = 88)			0.765
Serotypes 1 or 4	4 (19.0)	25 (18.9)	
Serotypes 2 or 3	11 (52.4)	48 (36.4)	
Known serological response to infection (*n* = 144)			0.551
Primary dengue infection	1 (4.8)	4 (3.0)	
Secondary dengue infection	20 (95.2)	119 (90.2)	
*Complete blood counts*			
Hemoglobin, median (IQR), g/dL	14.0 (12.5–16.6)	13.8 (12.8–14.9)	0.627
Hematocrit, median (IQR), %	41.7 (36.7–48.5)	41.1 (38.3–44.4)	0.934
WBC, median (IQR), ×10^3^ cells per μL	4.7 (2.4–10.5)	3.1 (2.3–4.6)	0.034
Absolute band form, median (IQR), cells per μL	188 (87–372)	142 (60–666)	0.237
Absolute PMN, median (IQR), cells per μL	2320 (1210–4126)	1620 (873–2264)	0.055
Absolute LYM, median (IQR), cells per μL	940 (405–2011)	758 (546–1126)	0.509
Absolute ALYM, median (IQR), cells per μL	684 (108–1370)	138 (68–379)	0.011
Platelet counts, median (IQR) × 10^3^ per μL	56.0 (12.0–71.0)	85.0 (56.0–136.2)	<0.001
*Categorical data*			
Hematocrit			0.928
≤ 2 % above reference range	7 (33.3)	49 (37.1)	
> 2 % above reference range	14 (66.7)	83 (62.9)	
Absolute band form			1.000
≤ 250 cells per μL	15 (71.4)	97 (73.5)	
> 250 cells per μL	6 (28.6)	35 (26.5)	
Absolute PMN			0.422
< 1500 cells per μL	7 (33.3)	60 (45.5)	
≥ 1500 cells per μL	14 (66.7)	72 (54.5)	
Absolute LYM			0.036
≤ 2000 cells per μL	16 (76.2)	122 (92.4)	
> 2000 cells per μL	5 (23.8)	10 (7.6)	
Absolute ALYM			0.002
≤ 300 cells per μL	8 (38.1)	99 (75.0)	
> 300 cells per μL	13 (61.9)	33 (25.0)	
Platelet counts			0.031
≤ 100 × 10^3^ per μL	18 (85.7)	77 (58.3)	
> 100 × 10^3^ per μL	3 (14.3)	55 (41.7)	
*Blood chemistries*			
Creatinine, median (IQR), mg/dL	0.8 (0.6–1.1)	0.8 (0.6–1.0)	0.739
Lactate level, median (IQR), mmol/L	2.7 (1.7–3.1)	1.4 (1.2–1.8)	<0.001
Albumin, median (IQR), g/dL	3.7 (3.2–4.3)	4.3 (4.0–4.5)	<0.001
AST, median (IQR), IU/L	219 (94–692)	76 (37–158)	<0.001
ALT, median (IQR), IU/L	100 (61–346)	47 (18–106)	0.003
*Categorical data*			
Lactate level			<0.001
< 2.0 mmol/L	7 (33.3)	111 (84.1)	
≥ 2.0 mmol/L	14 (66.7)	21 (15.9)	
Albumin			<0.001
< 3.5 g/dL	13 (61.9)	128 (97.0)	
≥ 3.5 g/dL	8 (38.1)	4 (3.0)	
AST			0.017
≤ 120 IU/L	7 (33.3)	84 (63.6)	
> 120 IU/L	14 (66.7)	48 (36.4)	
ALT			0.032
≤ 120 IU/L	11 (52.4)	102 (77.3)	
> 120 IU/L	10 (47.6)	30 (22.7)	

Abbreviations: *RT-PCR* reverse-transcriptase polymerase chain reaction, *IQR* interquartile range, *WBC* white blood cell counts, *PMN* neutrophils, *LYM* lymphocytes, *ALYM* atypical lymphocytes, *AST* aspartate aminotranferase, *ALT* alanine aminotransferase

### Univariate and multivariate analyses for development of severe dengue

A univariate logistic regression analysis was used to determine which of the baseline characteristics, clinical parameters, and laboratory findings may be associated with the development of severe dengue. All clinical factors potentially associated with the development of severe dengue were included in the univariate logistic regression analysis. We identified the following clinical factors associated with severe dengue: (1) age >40 years, (2) receiving NSAIDs or COX II inhibitors before admission, (3) persistent vomiting, (4) absolute lymphocytes >2000 cells per μL, (5) absolute atypical lymphocytes >300 cells per μL, and (6) lactate level ≥2.0 mmol/L (Table 3).

**Table 3:** Regression analyses. Univariate and multivariate logistic regression analysis of baseline characteristic, clinical and laboratory parameters for development of severe dengue according to the WHO’s 2009 definition among 153 hospitalized adults with dengue

Characteristics	Univariate logistic regression analysis		Multivariate logistic regression analysis	
	Odds ratio (95 % CI)	*p*-value	Odds ratio (95 % CI)	*p*-value
Age				
≤ 40 years	1.000 (Reference)		1.000 (Reference)	
> 40 years	4.461 (1.655–12.023)	0.003	5.215 (1.538–17.689)	0.008
NSAIDs or COX II inhibitors				
No	1.000 (Reference)			
Yes	3.662 (1.211–11.070)	0.021		
Persistent vomiting				
No	1.000 (Reference)		1.000 (Reference)	
Yes	5.850 (2.114–16.187)	0.001	4.817 (1.375–16.873)	0.014
Absolute lymphocytes				
≤ 2000 cells per μL	1.000 (Reference)			
> 2000 cells per μL	3.812 (1.156–12.574)	0.028		
Absolute atypical lymphocytes				
≤ 300 cells per μL	1.000 (Reference)		1.000 (Reference)	
> 300 cells per μL	4.875 (1.858–12.794)	0.001	3.163 (1.017–9.834)	0.047
Lactate level				
< 2.0 mmol/L	1.000 (Reference)		1.000 (Reference)	
≥ 2.0 mmol/L	10.571 (3.811–29.321)	<0.001	7.340 (2.334–23.087)	0.001

Abbreviations: *CI* confidence interval, *NSAIDs* nonsteroidal anti-inflammatory drugs, COX II cyclooxygenase II

All parameters with *p* ≤ 0.2 in the univariate logistic regression analysis were then further analyzed by a stepwise multiple logistic regression using the backward selection method in order to determine the independent clinical factors significantly associated with the development of severe dengue. We found the following clinical and laboratory findings to be independently associated with the development of severe dengue: (1) age >40 years (odds ratio [OR]: 5.215; CI: 1.538–17.689; *p* = 0.008), (2) persistent vomiting (OR: 4.817, CI: 1.375–16.873; *p* = 0.014), (3) absolute atypical lymphocyte >300 cells per μL (OR: 3.163, CI: 1.017–9.834; *p* = 0.047), and (4) lactate level ≥2.0 mmol/L (OR: 7.340, CI: 2.334–23.087; *p* = 0.001) (Table 3).

### Lactate level and absolute atypical lymphocytes by dengue severity

**Fig. 2:**
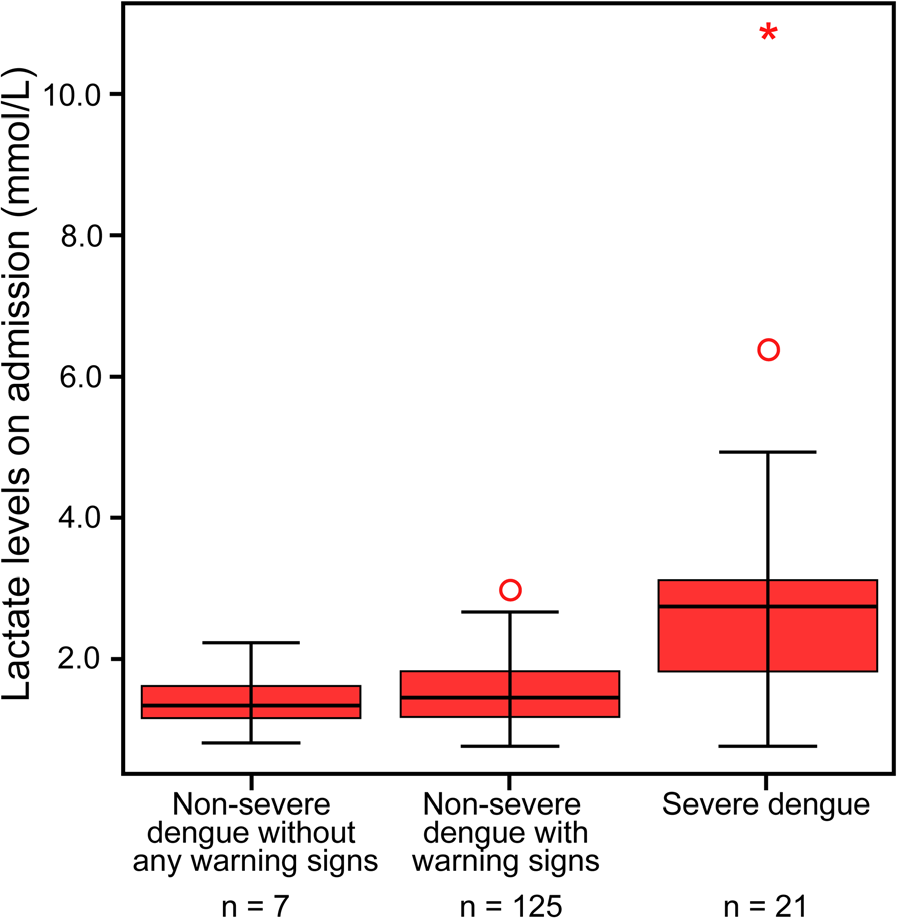
Lactate levels at admission. Distribution of lactate levels at admission of 153 hospitalized adults with severe dengue and non-severe dengue with and without warning signs (according to the WHO’s 2009 definition). Non-severe dengue without warning signs was not significantly different than non-severe with warning signs (*p* = 0.490). Severe dengue was significantly different than both non-severe with warning signs (*p* < 0.001) and non-severe without warning signs (*p* = 0.006)

**Fig. 3:**
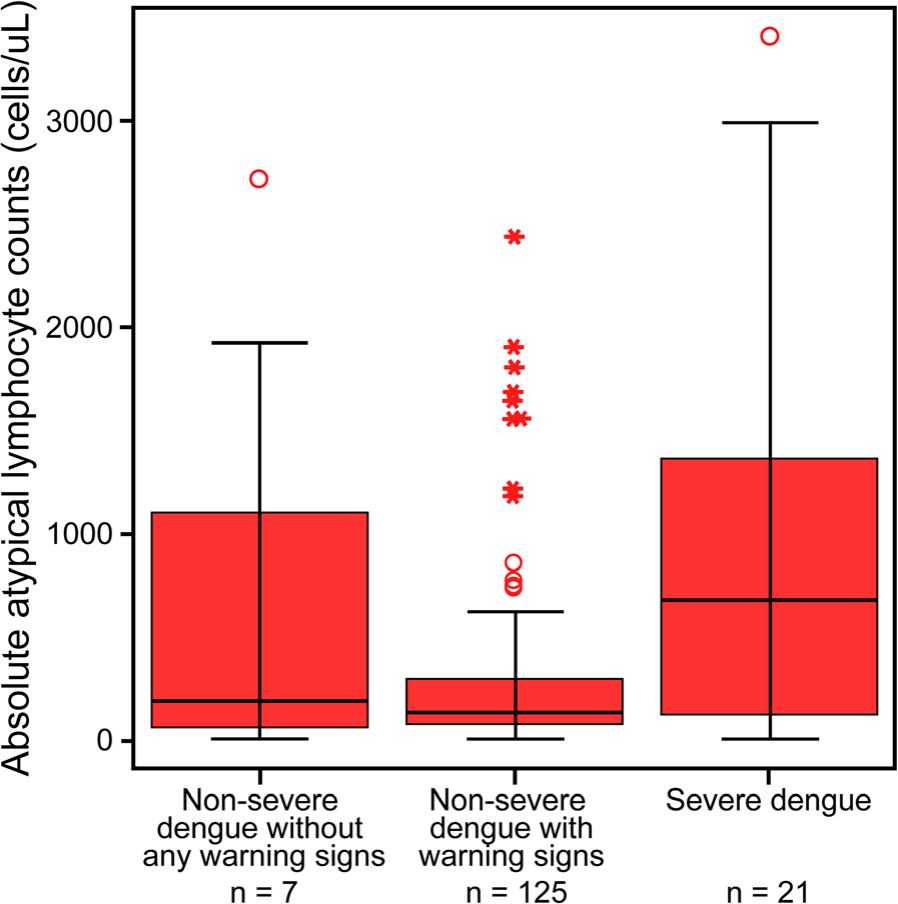
Absolute atypical lymphocyte levels. Distribution of absolute atypical lymphocytes among 153 hospitalized adults with severe dengue and non-severe dengue with and without warning signs (according to the WHO’s 2009 definition). Non-severe dengue without warning signs was not significantly different than non-severe with warning signs (*p* = 0.859). Severe dengue was significantly different than non-severe with warning signs (*p* = 0.016), but not non-severe without warning signs (*p* = 0.533)

The median (IQR) lactate levels in patients with dengue are shown in Fig. 2. The level of lactate among patients with severe dengue was significantly greater than patients with non-severe dengue (*p* < 0.05) and the extent of elevated lactate was associated with severity of dengue. Similarly, atypical lymphocyte count also increased with severity of dengue (Fig. 3) and patients with severe dengue had significantly higher levels of atypical lymphocytes than those with non-severe dengue with warning signs (*p* = 0.016).

## Discussion

Dengue is the most common mosquito-borne viral disease in humans and it is a significant public health problem, particularly in Asian-Pacific regions like Thailand [1]. Previous reports have shown that both the number of adults infected with dengue and its severity have increased dramatically in recent decades [2, 4–7] and the clinical factors associated with severe dengue in adults have varied widely between studies [10–14]. We therefore conducted a prospective study among Thai adults admitted to the hospital with dengue in order to identify the clinical factors associated with severe dengue according to the WHO’s 2009 definition, which would help improve the diagnostic process of severe dengue.

The majority of hospitalized adults with dengue had a secondary dengue infection. We found no association between secondary infection and the development of severe dengue, consistent with previous studies [20, 21]. However, a number of reports showed that the secondary dengue infection was associated with severe dengue [11, 13, 22]. Furthermore, in contrast to previous studies showing that DENV 2 and DENV 3 were associated with the occurrence of severe dengue [23, 24], we found no association between dengue serotypes and disease severity. Indeed, a recent study from Thailand showed that, rather than dengue serotypes, a longer period of time between sequential DENV infections was associated with more severe infections, a finding supported by the role of heterotypic immunity in either protection or enhancement of the infection [25].

With regard to the WHO’s 2009 guidelines for the management of dengue, patients with warning signs who develop high fever should be administered acetaminophen along with tepid sponge application for reducing the fever; however, NSAIDs should be avoided as these drugs may aggravate the complications, particularly gastrointestinal tract bleeding [8]. At present, it is well established that the use of NSAIDs increase the risk of gastrointestinal, renal, and cardiovascular side effects [26, 27]. COX II inhibitors have been shown to reduce gastrointestinal side effects, although cardiovascular and renal side effects still develop [27, 28]. A previous report from the Philippines showed that, of 24 American military personnel who were hospitalized with dengue, 1 developed dengue shock syndrome and had upper gastrointestinal bleeding; however, this patient most likely had a history of aspirin use [29]. However, a recent study showed that aspirin could suppress flavivirus replication [30]. Our univariate analysis indicated that patients who received NSAIDs or COX II inhibitors were more likely to develop severe dengue, but that the use of NSAIDs or COX II inhibitors was not independently associated with the development of severe dengue.

In our study, multivariate regression indicated the following clinical and laboratory characteristics upon admission were independently associated with the development of severe dengue: (1) age >40 years, (2) persistent vomiting, (3) absolute atypical lymphocyte >300 cells per μL, and (4) lactate level ≥2.0 mmol/L. In addition, patients with severe dengue commonly presented with severe plasma leakage and severe organ involvement. Age >40 years was independently associated with severe dengue in our study, consistent with a previous retrospective analysis from France, which reported that plasma leakage was the most common presentation for adults with severe dengue and that an age of >37 years predicted plasma leakage [14]. Our results also suggest that persistent vomiting at admission could be used to predict the development of severe dengue, which is similar to a finding of a previous multicenter study showing that persistent vomiting was one of the warning signs for severe dengue [31]. Although the sensitivity of persistent vomiting for identifying severe dengue in adults was very low (6.0–23.0 %), the specificity was as high as 93.0–96.0 % and the negative predictive value was 82.0–97.0 % [32, 33].

Regarding laboratory findings on admission, patients having >300 cells/μL of absolute atypical lymphocytes could be used to predict the development of severe dengue as patients with severe dengue had significantly greater levels of absolute atypical lymphocytes than patients with non-severe dengue. These findings are similar to those of a previous Thai study showing that atypical lymphocytes among patients with dengue were correlated with the presence of CD19 + B lymphocytes [34]. After a secondary dengue infection, atypical lymphocytes could indicate an augmented immune response attempting to control the spread of dengue-infected cells [35]. Simultaneously, these antibodies could enhance the entry of the dengue virus into macrophages and dendritic cells whereupon the virus would replicate [36]. Previous reports have also indicated that patients with higher dengue viremia have higher disease severity [37].

In addition, patients having lactate levels ≥2.0 mmol/L could be used as a predictor of the development of severe dengue as elevated lactate levels was observed to correspond to dengue severity in our study. Currently, lactate is used as a biomarker for severity of systemic hypoperfusion or impaired microcirculatory perfusion regardless of organ failure and shock [38]. One previous report has also indicated that elevated arterial or central venous lactate levels could be used to predict in-hospital mortality associated with many conditions such as infection or sepsis, liver disease, trauma, or cardiac arrest [39]. However, the arterial or central venous sampling required to measure lactate concentration is invasive and may increase risk of bleeding in patients with dengue. One previous study has shown a strong correlation between arterial and peripheral venous lactate levels (*r*^*2*^ = 0.89) [40], and so we used peripheral venous sampling for lactate concentration. We found that the presence of peripheral venous lactate ≥2.0 mmol/L at admission was independently associated with severe dengue. This finding is similar to that of a previous study among children with dengue [41]. We also show that peripheral venous lactate levels at admission are associated with increasing dengue severity in a dose-dependent manner. MAP and pulse pressure at admission were not significantly different among patients with severe and non-severe dengue. Previous studies have suggested that microvascular leakage occurs among patients with severe dengue leading to systemic hypoperfusion or impaired microcirculatory perfusion that can progress to shock [36, 42, 43]. These studies have suggested that systemic hypoperfusion or impaired microcirculatory perfusion in dengue probably occur prior to decreases in blood pressure and other hemodynamic parameters. Another possibility is that blood pressure and pulse pressure are insensitive hemodynamic parameters for identifying systemic hypoperfusion or microcirculatory failure among patients with dengue. In our study, lactate levels at admission could be used as a predictor for the severity of dengue. However, further studies are essential to assess the performance of lactate level as a diagnostic biomarker and/or its combination with other associated clinical factors for identifying severe dengue.

Our study has some limitations: (1) we recruited patients from only a single center in Thailand and our data may not be representative of dengue patients elsewhere, (2) only hospitalized patients were recruited to this study and we included few patients with non-severe dengue without warning signs, and (3) patients were admitted to the hospital at different times following the onset of fever and so the exact time of elevated level of lactate in patients with severe dengue could not be determined.

## Conclusions

Our findings indicate that age (>40 years), persistent vomiting, absolute atypical lymphocytes (>300 cells per μL), and lactate level (≥2.0 mmol/L) at admission could be used to identify those individuals at greatest risk of developing severe dengue. Early recognition and prompt management of adults with severe dengue might help to reduce morbidity and mortality in future.

## Abbreviations

ALT: Alanine aminotransferase
AST: Aspartate aminotransferase
ALYM: Atypical lymphocytes
CI: Confidence interval
COX II inhibitors: Cyclooxygenase II inhibitors
DENV: Dengue virus
DHF: Dengue hemorrhagic fever
ELISA: Enzyme-linked immunosorbent assay
HR: Heart rate
IQR: Inter-quartile range
JEV: Japanese encephalitis virus
LYM: Lymphocytes
MAP: Mean arterial pressure
NSAIDs: Nonsteroidal anti-inflammatory drugs
OR: Odds ratio
PMN: Neutrophils
PP: Pulse pressure
RR: Respiratory rate
RT-PCR: Reverse-transcriptase polymerase chain reaction
STROBE: Standards for the reporting of observation studies in epidemiology
WBC: White blood cell counts
WHO: World health organization
